# Encapsulation of Fiber Optic Sensors in 3D Printed Packages for Use in Civil Engineering Applications: A Preliminary Study

**DOI:** 10.3390/s19071689

**Published:** 2019-04-09

**Authors:** Richard Scott, Miodrag Vidakovic, Sanjay Chikermane, Brett McKinley, Tong Sun, Pradipta Banerji, Kenneth Grattan

**Affiliations:** 1 School of Mathematics, Computer Science & Engineering, University of London, London EC1V 0HB, UK; miodrag.vidakovic.1@city.ac.uk (M.V.); b.mckinley@city.ac.uk (B.M.); t.sun@city.ac.uk (T.S.); k.t.v.grattan@city.ac.uk (K.G.); 2 Department of Civil Engineering, Indian Institute of Technology Roorkee, Roorkee 247667, India; sanjay.chikermane@gmail.com (S.C.); pbanerji.iitb@gmail.com (P.B.)

**Keywords:** fiber optic sensor, encapsulation, 3D printing, civil engineering, strain measurement

## Abstract

Fiber optic sensors have considerable potential for measuring strains in the challenging environment posed by today’s civil engineering applications. Their long-term reliability and stability are particularly important attributes for assessing, with confidence, effects such as cracking and response to normal (and abnormal) loads. However, given the fragile nature of the bare fiber, the sensors must be packaged to achieve adequate robustness but the resulting increased cost of installation can frequently limit the number of sensors which can be installed or their use may have to be ruled out altogether due to these financial constraints. There is thus potential for the development of a more affordable type of packaging and this paper describes work undertaken to produce a cost-effective and easy-to-use technique for encapsulating fiber optic sensors in resin, taking advantage of 3D printing techniques which are widely available and at low cost. This approach can be used to produce a robust, inexpensive packaged sensor system which is seen as being suitable to be extended to a wider range of uses including installation in concrete structures prior to casting. To evaluate this approach, several such 3D printed package types and geometries are described and their behavior is assessed from a programme of laboratory trials, the results of which are presented in this paper. This proof-of-concept testing has demonstrated the considerable potential which 3D printed packages have and the scope for further development and consequent use in civil engineering applications. Areas showing promise and potential, which have been identified from the work undertaken, are discussed.

## 1. Introduction

There is an ongoing need to measure strains in reinforced concrete structures both in the laboratory and in the field. The former is mainly concerned with tests on structural elements such as beams, columns and slabs while the latter often involves monitoring full scale structures over extended time periods. Materials involved include concrete (both reinforced and prestressed, in situ and precast), steel, and timber. Concrete is highly alkaline and thus presents a harsh environment for any type of sensor and this, together with the rigors of sensor installation and the concrete casting process, means that sensors for concrete must be designed to be particularly robust and thus reliable, if they are to function correctly over long periods.

There are, potentially, many situations where optical fiber sensors could be used successfully in civil engineering applications because they are immune, or at least relatively immune when compared with other sensor types, to electromagnetic interference and moisture ingress. They also have the advantage of being small, compact and lightweight and have attracted considerable research interest in recent years [1,2,3,4,5,6]. Fiber Bragg Grating (FBG) sensors have been used successfully in a wide variety of civil engineering applications [7,8,9,10,11,12,13] but they are fragile and thus must be correctly packaged (i.e., encapsulated) in a way that is tailored to that environment to resist the effects of climate and usage, if they are to achieve the required level of robustness demanded in civil engineering environments. Additionally, they must be suitable for both mounting on the surface of a structure after construction and, in the case of reinforced/prestressed concrete structures, for inclusion in the structure prior to concreting. It is important to keep in mind the needs of practicing civil engineers—the end users—when developing a sensor system for field use since this is a far less controlled environment when compared to that found in the laboratory.

An in-fiber FBG is formed from a periodic modulation of the refractive index of the core of a photosensitive fiber where the modulation of the refractive index is induced by UV light from a laser source. The periodic modulation acts as a filter reflecting one wavelength, the Bragg wavelength (λ), which is expressed by the following formula [14]:

(1) $$\lambda =2n_{e}\Lambda$$

where *n_e_* is the effective refractive index and Λ is the period of the grating. Both strain and temperature changes will induce a shift of the Bragg wavelength, which can be modelled by the following equation:

(2) $$\Delta \lambda =S_{strain}\Delta \varepsilon +S_{T}\Delta T$$

where *S_strain_* and *S_T_* are the strain and temperature sensitivities, respectively and Δε and ΔT are the strain and temperature variations respectively. Equation 2 highlights the sensor temperature dependence associated with the strain measurement, particularly as *S_T_* is considerably larger than *S_strain_*. To have a meaningful determination of the actual (i.e., mechanical) strain, it is necessary to have an accurate value of the temperature in the vicinity of the FBG.

**Figure 1:**
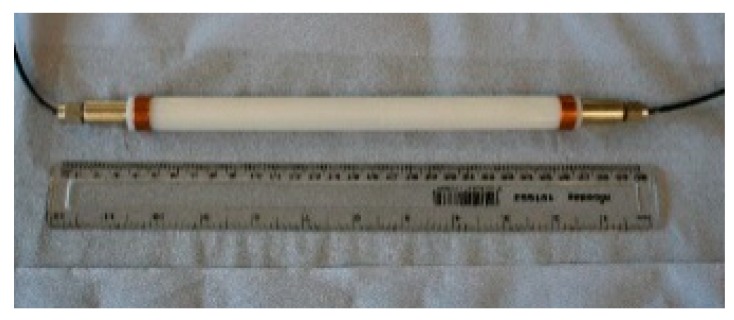
Commercial optical fiber sensor.

**Figure 2:**
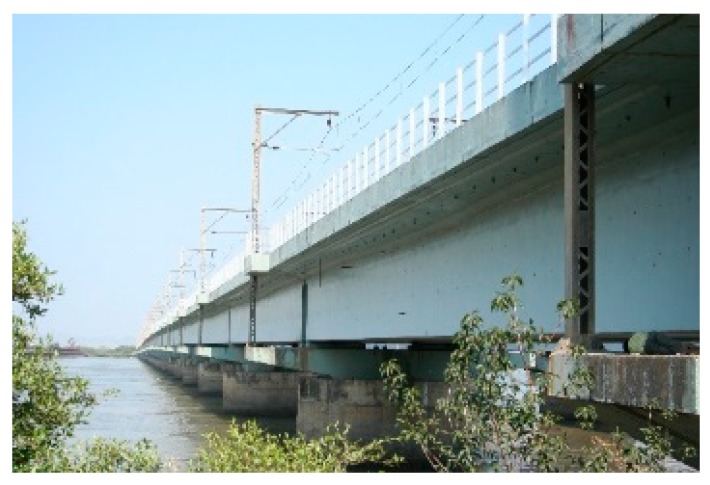
Mumbai railway bridge.

**Figure 3:**
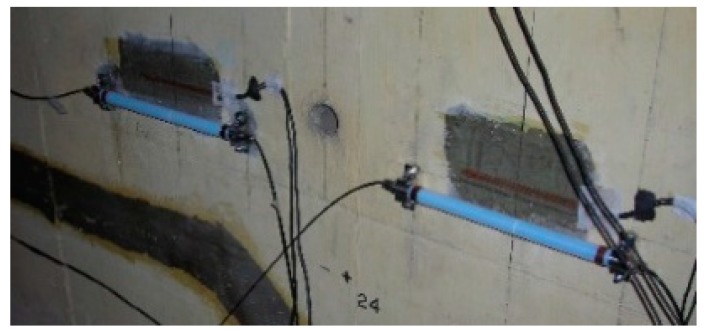
Sensors on side wall.

**Figure 4:**
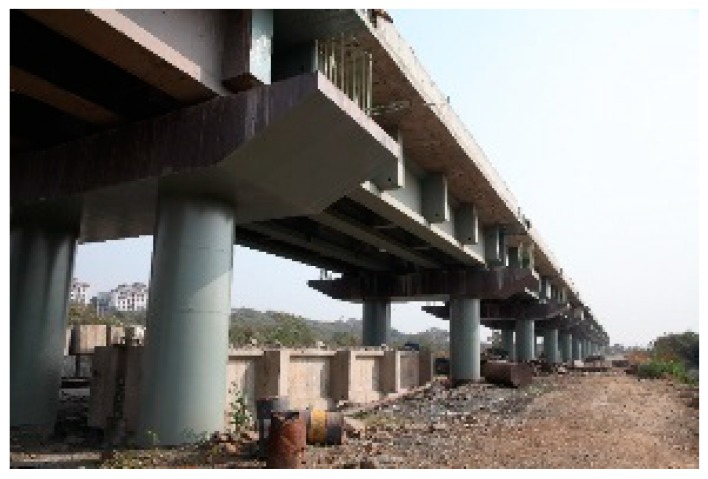
Mumbai railway viaduct.

**Figure 5:**
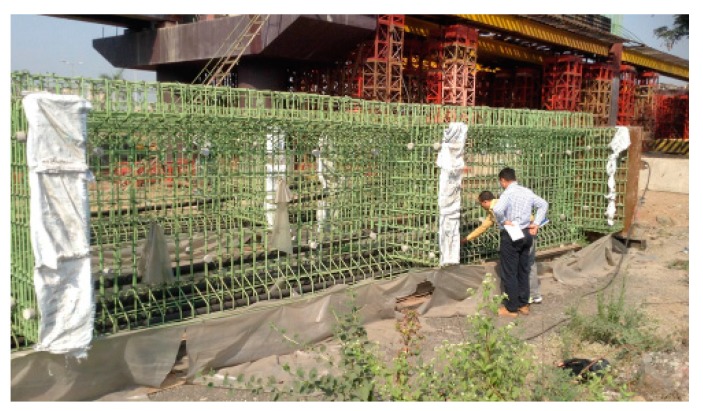
Sensors on reinforcement cage.

Commercial sensors are readily available for civil engineering applications and a model used by the authors is shown in Figure 1. It contains two FBGs, one measuring total (i.e., mechanical plus temperature) strain and the other measuring temperature strain, thus allowing the mechanical strain to be easily calculated. In a recent illustration of the use of packaged sensors for civil engineering-based strain measurement, six of these sensors were surface mounted by the authors on the walls of an existing prestressed concrete box girder railway bridge in Mumbai (Figure 2 and Figure 3) for monitoring the effects of passing trains [15] and sensors have recently been cast into a railway viaduct currently under construction, also in Mumbai (Figure 4 and Figure 5). Unfortunately, the extent of these installations to monitor the bridge widely has been severely constrained by the high price of the sensors as each cost several hundred US dollars and for a full assessment of a structure of this size, many hundreds would be needed. This problem can be overcome with more inexpensive devices and there is a potential market for a reliable, robust, low cost, packaged optical fiber sensor that can be used in this sort of environment, and this paper reports work to date by the authors to develop such a product.

Experience gained by the authors from their prior field work in India indicated that for a package to be successful it must be affordable, robust and durable, yet easy to produce in a range of geometries. The completed sensor also had to produce high quality, repeatable measurements, be suitable for surface mounting and also be able to withstand the harsh treatment that comes with being cast into concrete.

Over the last few years there have been considerable advances in the use of 3D printing techniques with both the hardware and software becoming much more affordable and this forms the basis of the low-cost sensor discussed. Since both were already available to the authors, a promising way forward that was identified was to fabricate a suitable packaging that was compatible with the fiber optic itself using 3D printing techniques. The work built on an initial trial carried out by the authors that was deemed sufficiently encouraging [16] to warrant the further work reported in this paper, through a collaboration between City, University of London, and the Indian Institute of Technology Roorkee.

## 2. Materials and Methods

**Figure 6:**
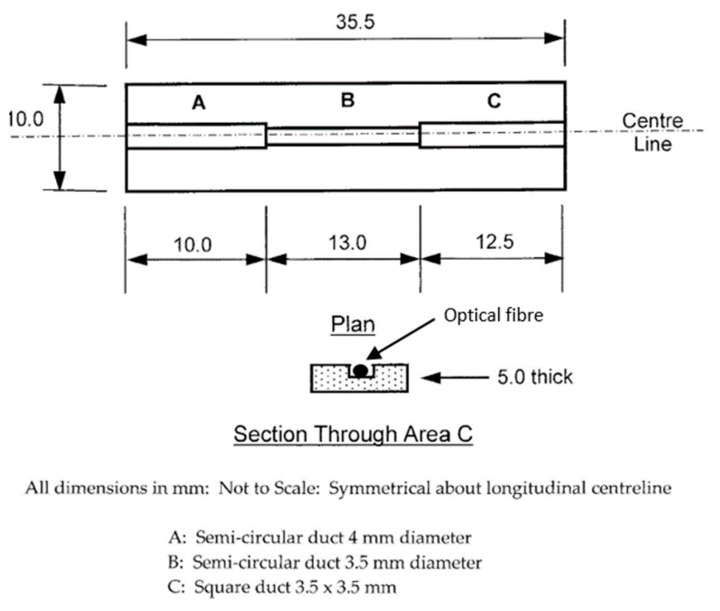
Layout of open-top package.

An approach that was both simple and robust was the basis for the design, where initially two similar open-top packages, as illustrated schematically in Figure 6, were designed with the use of the software package, SolidWorks. (The package was printed first followed by installation of the optical fiber as described below). Standard photopolymer resin was chosen for the fabrication process of the package, supplied by Formlabs. It is a high-resolution resin that ensures both high strength and high precision of the 3D printed parts, with a Young’s modulus of 1.7 GPa. Further, Formlabs Pre Form software was used to drive a Formlabs 1+ 3D printer after the model designed in SolidWorks was loaded. After 3D printing was completed, the samples were removed from the building platform and carefully rinsed in isopropyl alcohol (IPA). To achieve higher strength of the sample, all samples were post-cured under a UV (405 nm) lamp for 2 hours. After completion of the fabrication process, two 5 mm long FBGs were installed in each package, the one in the center of Section B being glued to the package with Duralco 4525-IP for strain measurement and the other free (i.e., not glued) in Section C for temperature measurement, both being multiplexed on a single fiber. The cable leading to the interrogator exited the package along Section A (see Figure 6) and protection of the strain FBG was achieved by filling Section B with Duralco adhesive. The temperature FBG was allowed to float in Section C (as it was not required to stabilize it further).

**Figure 7:**
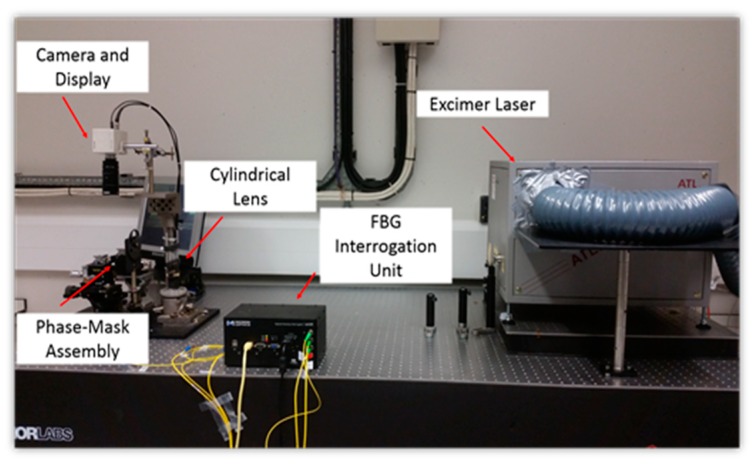
Basic configuration of FBG manufacturing.

**Figure 8:**
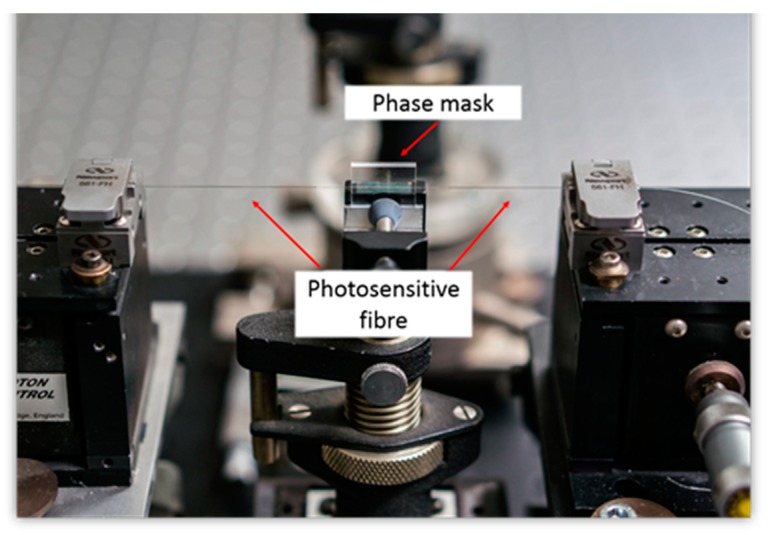
Phase mask setup for FBG manufacturing.

The FBGs used in this work as the basis of the sensor system were manufactured using an excimer laser-based FBG-fabrication facility at City, University of London. Boron/Germanium co-doped fibers from Fibercore (PS1250/1500) were used as the photosensitive fibers in which was imprinted an interference pattern, created by using a phase mask, after exposure to light from a high power KrF excimer laser, operating in the ultra violet at 248 nm. During the inscription process, the laser source operated with a pulse energy of 10 mJ and a pulse frequency of 100 Hz. Different phase masks were used in order to manufacture FBG sensors, with different Bragg wavelengths, which then allowed ease of multiplexing (and thus the identification of an individual grating by its signature wavelength) when wavelength-division-multiplexing method was used to take multiple measurements. A plano-cylindrical lens with a focal length of 200 mm was placed in front of the laser allowing the laser beam to be focused into a thin line, with a width of approximately 0.5 mm and length of around 8 mm. The laser beam was projected to the phase mask to create the required interference pattern which was subsequently imprinted to the photosensitive fiber which was placed close to the phase mask to modulate its core refractive index. To monitor closely the manufacturing process and control the reflectivity of the FBG fabricated, it was essential to monitor the FBG formation as it occurred during laser beam exposure by connecting it to a commercial interrogator system, Type *sm125* manufactured by Micron Optics, this being done using an external fiber. Figure 7 and Figure 8 show photographs of the equipment used and the way the gratings are fabricated for use in this sensor system.

Testing of the quality and integrity of the packaged sensors that were developed for this application was done by mounting them on a steel beam and then subjecting them to a series of load cycles in the well-calibrated laboratory environment. This approach of using a steel beam had the key advantage that its behavior would be linearly elastic under repeated loads provided, of course, that stresses were kept within the elastic range (as was ensured). A square hollow section was chosen as this type has excellent resistance to both lateral torsional buckling and web buckling.

**Figure 9:**
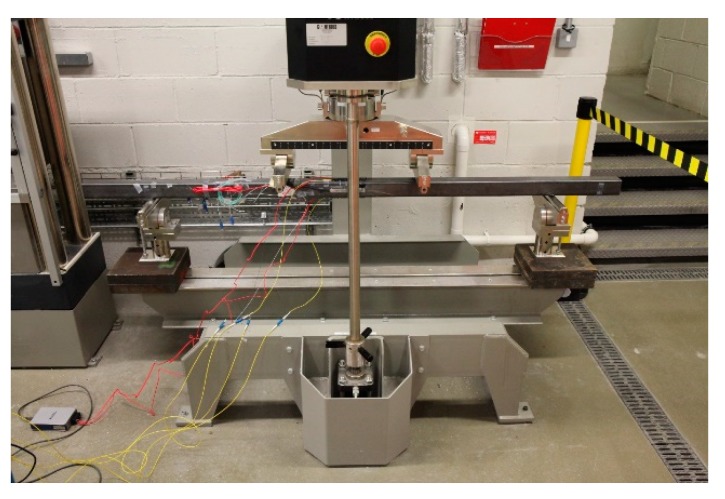
Test rig used in this work for assessment of the packaged sensors developed.

Therefore, the chosen test section for mounting the sensor package was a 60 mm × 60 mm square hollow section steel beam having a 3 mm wall thickness (i.e., a 60 × 60 × 3 SHS), which was mounted in a standard laboratory testing machine. The distance between the simple supports of the machine was 1500 mm and two point loads were applied using a spreader beam which gave a symmetrical four point loading arrangement. The constant moment zone (i.e., the distance between the two applied loads) was 500 mm, as is illustrated in Figure 9.

**Figure 10:**
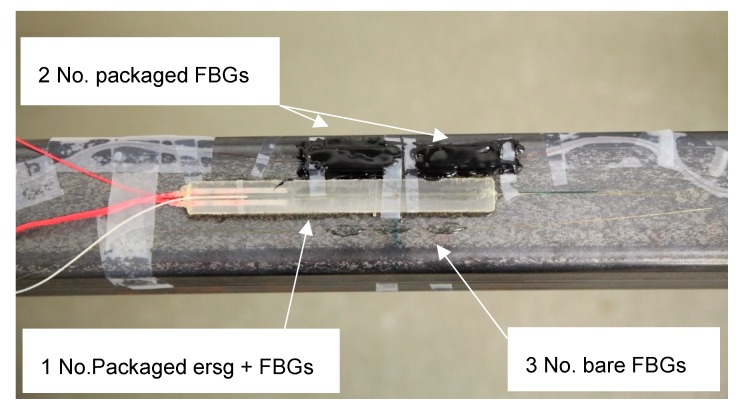
Detail of sensor layout on steel beam.

The two open-top packages were glued to the mid-point of the beam i.e., at the center of the constant moment zone and beside them a commercially sourced and packaged electric resistance strain gauge (esrg) was glued to provide a simple calibration—this being 125 mm long, 13 mm wide and 5 mm thick, with a Gauge Resistance of 120 ष and a Gauge Factor of 2.1 (Type PML-120-2LJD manufactured by Tokyo Measuring Instruments Lab). Duralco 4525-IPadhesive was used to bond all the sensor types to the steel beam, with particular care and attention being given to this operation in view of the importance of achieving full strain transfer between the bonded surfaces. Prior to mounting the packaged ersg sensor, a longitudinal groove was carefully cut in both the top and bottom faces of the packaging material into each of which were glued three bare FBGs multiplexed on a single fiber. Beside this packaged esrg, three bare FBGs, again multiplexed on a single fiber, were glued directly onto the face of the steel beam. This was done to allow the output data from the packaged FBGs to be benchmarked against all the other sensors (which then provided a high degree of redundancy, as is needed for use in-the-field). Figure 10 shows a photograph of this sensor arrangement used here.

To investigate the performance in detail, a series of load histories, representative of what would be experienced in-the-field, was applied to the beam to test the performance of the sensor system package under both cyclic and sustained loads. Data from the FBG-based sensors were collected using a Micron Optics Type *sm130* interrogator with a sampling frequency of 1 kHz and a wavelength accuracy of ±2 pm. This interrogator was chosen as it had 16 input channels, which enabled FBG-based sensors which used similar wavelengths to be kept as separate channels in this experiment, for ease of identification. Data from the ersg that was used for the cross-calibration were collected using a standard laboratory instrument.

**Figure 11:**
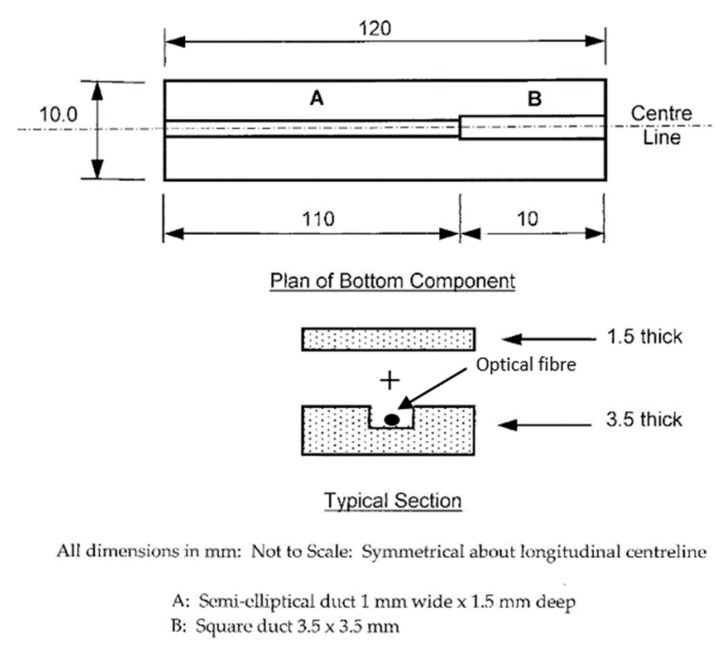
Layout of closed package.

**Figure 12:**
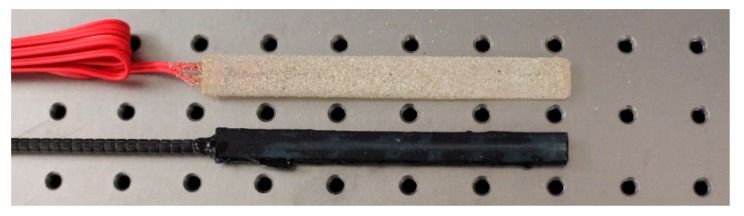
Packaged sensors: ersg top, FBG bottom.

Following on from these tests, results from which are discussed in the next section, a more sophisticated packaged sensor was designed and tested to allow for wider use in-the-field. The new sensor had similar overall dimensions to the packaged ersg sensors considered and used earlier, an approach which was designed to give confidence to users in industry when seeking to replace ‘familiar’ devices by new technology. The essential novelty of the work lies in the approach taken to 3D print sensor packages tailored to the specific need of the application and allow the incorporation of as many FBG-based sensors as are required for the specific use of the sensor package by industry. This allows the user to break away from the constraints of the use of conventional packaged esrgs. Since packaged ersgs are specifically designed for both surface mounting and embedment in concrete structures (without the need for bolted connections), it seemed sensible, for this exercise, to manufacture the new FBG-based sensor package to have similar dimensions and surface characteristics for easy comparisons to be made. This shows the versatility of the approach used. However, in other applications the sensor package could be designed to be completely different from that where esrgs are used and be lighter and more compact, or contain a larger number of sensors. Such flexibility in design with the FBGs, the 3D printing and an ability to meet the specific needs of the geometry and conditions of the site where the packaged device is to be used is a strength of this approach, described here for one such specific application. The device thus designed is shown in Figure 11, it being printed in two parts, with the dimensions given in the figure. Here the package consisted of a *top* and a *bottom*, where these two parts were glued together after the installation of the FBGs that formed the sensor elements themselves. The device was made more robust as a result of this design, with a view for it being cast in a concrete beam and yet survive to read-out the strain data. To assist with this, a tough photopolymer resin, cured under UV light, was used which operated at a temperature between 40 to 50 °C. Curing lasted for one hour, and when tested the Young’s modulus was 2.5 GPa. Again, Duralco 4525-IP resin was used to bond together the two halves of the package. Two FBGs were again installed in each package, mirroring the previous design. The FBG in Section A (to be used for strain measurement) was glued to the package with Duralco 4525-IP while the FBG in Section B (to be used for temperature measurement) was kept free by not being glued. Again, both FBGs were multiplexed onto a single fiber. Figure 12 shows the similarity in the dimensions of the commercial packaged ersg and the system created using the FBG sensors described (this being done to give user confidence in ease of *switching* between one technology and the other). The FBG-based sensor package was evaluated in the laboratory using a similar sensor layout and test procedure to that described above for the previous design.

## 3. Results

### 3.1. Open-Top Packages

Very similar responses were obtained from the packaged ersg and the bare FBGs that were evaluated, and this was the case with all the load histories examined, with all showing excellent linearity and repeatability, with a total absence of creep. The temperature-monitoring FBG in the packaged sensor was stable throughout the tests, indicating that no significant temperature change had occurred (and thus no corrections for temperature were required).

**Figure 13:**
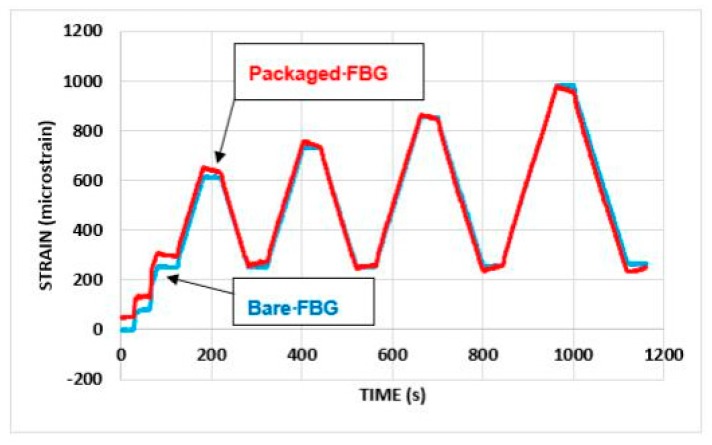
Open-top package: response to load cycles to 5.0, 6.0, 7.0 and 8.0 kN (total load).

**Figure 14:**
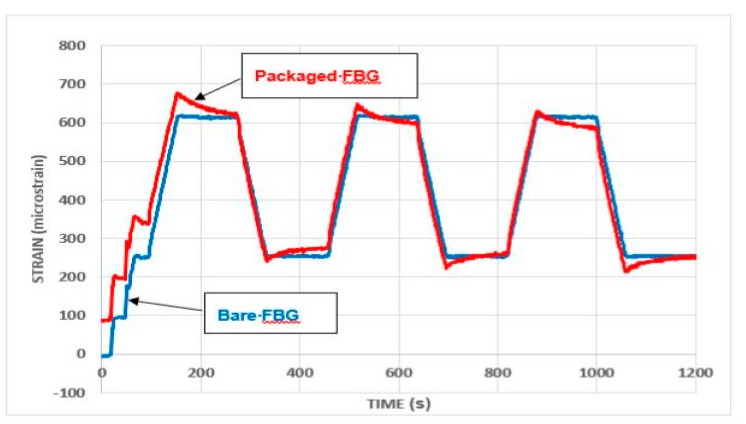
Open-top package: repeated load cycles to 5.0 kN (total load).

Figure 13 and Figure 14 show a comparison of the behavior of the strain monitoring FBG when configured in the packaged sensor design, with that from the bare FBGs. Here Figure 13 shows results for successive increases in total load on the beam, while Figure 14 shows the behavior for repeated load cycles, with a total load of up to 5 kN being applied.

On first loading, the strain responses from the packaged sensor were greater than of those from the bare FBGs, possibly due to an initial *bedding-in* of the package on the steel beam, but this effect became less marked as the load was increased. The measurement of strains during both the loading and unloading was generally very similar, with the most noticeable feature of the results being the pronounced reduction in strain when loads were sustained (as is illustrated in Figure 14). This reduction was most likely caused by creep, but local slip or loss of adhesion between the package and the beam may also have occurred.

### 3.2. Closed Top Package

The FBG used for temperature monitoring in the packaged sensor was again stable throughout the tests (and thus no temperature corrections were needed).

**Figure 15:**
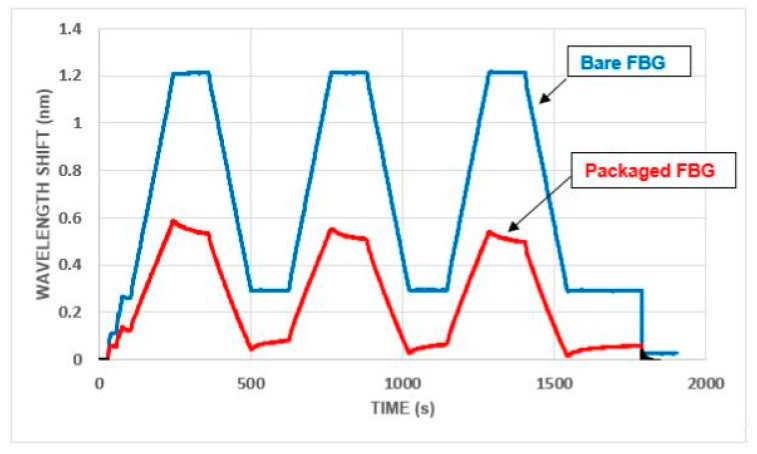
Closed top packages: repeated load cycles to 5.0 kN (total load).

**Figure 16:**
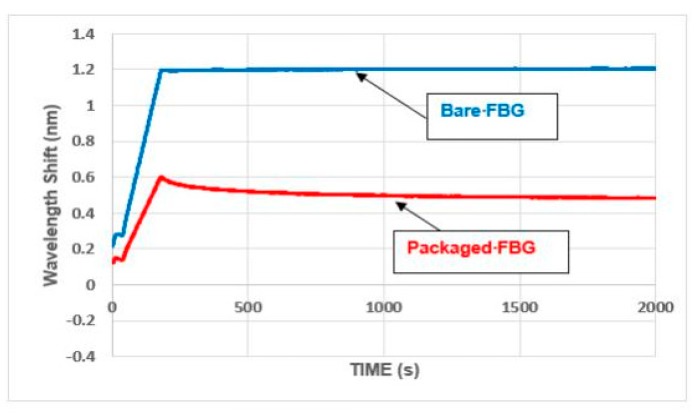
Closed top package: creep behavior at 5.0 kN (total load).

Overall, as before, the response of the strain monitoring FBG in the packaged sensor was similar to that for the bare FBGs. A typical comparison (in compression) is given in Figure 15. Using the wavelength shift for the vertical axis emphasizes that the stiffness of the packaged FBG was significantly less than that for the bare FBG (which is discussed in more detail in the next section). Creep effects were also still present, although these were less pronounced than that which occurred with the open-top packages and also died away quite rapidly (as can be seen from Figure 16).

## 4. Discussion

Strain sensitivities of the FBG-based packaged sensors were calculated by referencing them to the ersg results as a simple means of cross-calibration. The sensitivities of the FBG-based sensors used for temperature measurement were known from prior work but not explicitly calculated since, in view of the stable laboratory environment (in these tests temperature effects were minimal, and ignored), the focus was on the measured wavelength shifts which represent the underpinning sensor performance. Temperature calibration could be performed easily in future tests and would include controlled heating and cooling cycles. This would be particularly important for applications in-the-field where thermal effects are often unpredictable and thus accurate correction for temperature is needed. A specific example is the measurement of thermochemical effects during the period when concrete is curing. It is also recognized that polymer structures absorb water (i.e., swell) and the resulting effect on sensor performance will require some consideration.

The strain sensitivity of the open-top packaged FBG-based sensors was found to be significantly lower (0.68 pm/microstrain) than that for the bare FBGs (1.13 pm/microstrain). This lower sensitivity is not unexpected when sensors are packaged and the low stiffness of the resin used for the packaging was most likely the cause of the creep problem. This does not represent a problem in use as the packaged sensor is then calibrated, rather than the bare FBG itself. The slightly lower sensitivity seen means that this packaged device can be used for all but the most sensitive of measurements desired. 

The sensitivity of the closed top package was found to be 0.54 pm/microstrain in tension and 0.36 pm/microstrain in compression. Bearing in mind that a stiffer resin had been used for this sensor, the reduction in stiffness and the continuing presence of creep were both somewhat disappointing but again the behavior and overall sensitivity of the packaged sensor was still considered to be encouraging for use in-the-field.

Therefore, this slightly lower sensitivity, although undesirable, was not, in itself, seen as any real problem as the sensors of this type will be calibrated in advance of their use and the packaging conditions are consistent from sensor to sensor, thereby making the calibration predictable and consistent from device to device. Consequently, these tests were deemed sufficiently encouraging to justify further development work being undertaken.

It is recognized in the work described in this paper that this is just the first stage in the development of inexpensive, easy-to-use packaged fiber optic sensors which are suitable for commercial civil engineering applications and, given the packaged dimensions, to be direct replacements for packaged esrgs. Therefore, this early proof-of-concept work shows promise and gives encouragement for further development work to be undertaken. Experience gained from the work reported in this paper indicates that issues to be addressed further include the following:

- significantly reduce the mismatch between the stiffness of the packaging material of the sensors and that of concrete or steel, which is the likely root cause of the creep problems. Possibilities to overcome this include using PEEK (polyether ether ketone) or, perhaps more likely, ceramic resins for the packaging, although it is important to keep in mind the need to control costs to allow wider adoption of the sensor device. However, it is good to note that PEEK is not more overly expensive compared with the resins used to date but using ceramics requires more sophisticated hardware for the curing process. As often happens with engineering decisions, optimizing the sensor requires a trade-off between performance and cost, but costs would have to rise considerably before 3D-printed packages were as expensive as the currently available commercial sensors. Additionally, reducing the thickness of the packaging is highly desirable and may be assisted by encapsulating the FBGs in the packaging at the time of printing.
- perform durability tests of the package materials to assess resistance to an alkaline environment, moisture and wear to allow their use in a wide variety of environments.
- ensure sensors perform the same in tension and in compression, maximize the sensitivities (when compared to the figures achieved to date) and ensure that these are consistent in performance between sensors of a similar type and size.
- ensure similar behavior between sensors which are surface mounted and those which are embedded.

Finally, an important aspect of the next stage will be to evaluate sensor performance under more typical civil engineering conditions, such as embedment in reinforced concrete beams. Installation of sensors in a concrete beam must always be undertaken very carefully to preserve the integrity of the sensors and civil engineers are well experienced with this. Consequently, no additional problems are anticipated when installing packaged FBG sensors compared with the established packaged ersg sensors. Good performance is expected from the FBG packages and this will be reported in due course.

## 5. Conclusions

A number of conclusions can be drawn from the positive outcomes of the above work, as follows:

- a need for low-cost packaged fiber optic sensors for strain measurement in civil engineering applications has been identified and then met in the design reported, with potential for use in monitoring reinforced concrete structures.
- sensor systems of that type have been effectively packaged (encapsulated) in resin using 3D printing techniques, creating a low-cost and effective device for use in these applications which has a consistent calibration and good sensitivity.
- ‘proof-of-concept’ laboratory testing has demonstrated the potential of the packaged sensors for strain measurement in civil engineering applications.
